# A Novel Variant in Superoxide Dismutase 1 Gene (*p.V119M*) in Als Patients with Pure Lower Motor Neuron Presentation

**DOI:** 10.3390/genes12101544

**Published:** 2021-09-29

**Authors:** Claudia Ricci, Fabio Giannini, Giulia Riolo, Silvia Bocci, Stefania Casali, Stefania Battistini

**Affiliations:** Department of Medical, Surgical and Neurological Sciences, University of Siena, 53100 Siena, Italy; fbio.giannini@unisi.it (F.G.); giulia.riolo@gmail.com (G.R.); silvia.bocci87@gmail.com (S.B.); stefania.casali1@gmail.com (S.C.); stefania.battistini@unisi.it (S.B.)

**Keywords:** amyotrophic lateral sclerosis, *SOD1* gene, novel variant, *p.V119M*, pure lower motor neuron phenotype, flail leg variant, progressive muscular atrophy, phenotype-genotype correlation, modelization

## Abstract

Amyotrophic lateral sclerosis (ALS) is a progressive and fatal disorder characterized by degeneration of motor neurons in the cerebral cortex, brain stem, and spinal cord. Most cases of ALS appear sporadically, but 5–10% of patients have a family history of disease. Mutations in the superoxide dismutase 1 gene (*SOD1*) have been found in 12–23% of familial cases and in 1–2% of sporadic cases. Currently, more than 180 different *SOD1* gene variants have been identified in ALS patients. Here, we describe two apparently sporadic ALS patients carrying the same *SOD1* c.355G>A variant, leading to the *p.V119M* substitution, not previously described. Both the patients showed pure lower motor neuron phenotype. The former presented with the flail leg syndrome, a rare ALS variant, characterized by progressive distal onset weakness and atrophy of lower limbs, slow progression and better survival than typical ALS. The latter exhibited rapidly progressive weakness of upper and lower limbs, neither upper motor neuron nor bulbar involvement, and shorter survival than typical ALS. We provide an accurate description of the phenotype, and a bioinformatics analysis of the *p.V119M* variant on protein structure. This study may increase the knowledge about genotype-phenotype correlations in ALS and improve the approach to ALS patients.

## 1. Introduction

Amyotrophic Lateral Sclerosis (ALS) is an adult-onset, progressive and fatal neurodegenerative disease characterized by the degeneration of the motor neurons in the brain and spinal cord, resulting in progressive weakness of bulbar, thoracic, abdominal and limb muscles. Classical ALS (“Charcot ALS”) is defined by the selective deterioration of both upper motor neurons (UMN) and lower motor neuron (LMN) [1]. Damage of UMN in the motor cortex causes hyperreflexia, extensor plantar response and increased muscle tone, whereas dysfunction of LMN in the brainstem and spinal cord leads to generalized weakness, hyporeflexia, muscle atrophy, muscle cramps and fasciculations [2].

In the early stages, symptoms may be very different. At presentation, most affected individuals show spinal onset, characterized by focal limb weakness initiating in a distinct region of the body and then propagating from this area to secondary sites [3]. On the other hand, bulbar onset accounts for about 25% of ALS cases and is typically characterized by slurred speech and difficulty swallowing. In the majority of cases, limbs symptoms occur within 1–2 years. During the disease course, most cases become generalized and show combinations of both LMN and UMN signs affecting spinal and brainstem regions [4]. Other brain functions, such as oculomotor and sphincter functions, are rarely involved. Behavioural, cognitive or executive impairments have been reported in more than 30% of cases, while frontotemporal dementia (FTD) is described in 20.5% of patients [5]. Cognitive impairements may precede or occur after the onset of motor symptoms. Approximately 3% to 5% of patients show cognitive or respiratory onset [6]. Death is mainly due to respiratory failure, and occurs 2–4 years after onset, although a poor prognosis is often associated with bulbar and respiratory onset [7]. In a small percentage of patients disease duration may be longer (≥10 years).

Different ALS phenotypes have been recognised with distinct clinical presentations, progression and prognosis. Some of them are characterized by a predominant or selective LMN presentation, with marginal or absent UMN dysfunction. They include flail arm (FA), flail leg (FL) and progressive muscular atrophy (PMA) phenotypes [4,8]. Among these, the flail leg syndrome, also known as the Pseudopolyneuritic or the Marie-Patrikios form of ALS, is a rare condition (about 3–3.5% of all motor neuron disease cases) [9] characterized by distal and asymmetrical onset weakness and wasting of the lower limbs, where the disease remains long time confined. Flail-leg syndrome is thus described as a LMN variant, however UMN involvement can appear in the later stages of disease [10]. A slower progression and significantly better prognosis compared to more typical forms of ALS have been reported [10], with a median survival times of 5 to 6 years [11]. The longer FL is confined to the legs, the longer is survival [10]. The PMA phenotype is characterized by progressive LMN signs without clinical evidence of UMN involvement, although UMN signs may occur during the disease course. This phenotype represents ~ 5% of all motor neuron disease cases and is usually characterized by a relatively earlier age of onset, and a slower progression than typical ALS [12]. However, axial onset is often associated with rapid progression, early onset of respiratory failure and poor prognosis [13].

Approximately 90% of overall cases present as a sporadic form of ALS (SALS), whereas 5–10% have a positive family history and are referred as familial form of ALS (FALS), even though the true rate of familial disease is probably underestimated [14]. Mutations in known genes explain about 70% of familial cases and 10% of sporadic cases [15]. In Europe, the GGGGCC hexanucleotide repeat expansion in the chromosome 9 open reading frame 72 (*C9orf72*) gene is the most common genetic cause of FALS (33.7%) and SALS (5.1%), followed by mutations in the copper/zinc superoxide dismutase 1 (*SOD1*) gene (14.8% in FALS and 1.2% in SALS cases), TAR DNA binding protein (*TARDBP*) gene (4.2% and 0.8%, respectively), and FUS RNA binding protein (*FUS*) gene (2.8% and 0.3%) [15,16,17].

*SOD1* was the first gene demonstrated to be associated with ALS in 1993, related to an adult-onset autosomal dominant form of the disease (ALS1) [18]. The SOD1 protein is a homodimeric Cu/Zn-binding enzyme that catalyzes the conversion of the toxic superoxide anion (O^2−^) to hydrogen peroxide (H2O2) and molecular oxygen (O2). SOD1 is ubiquitously expressed and is found in the cytosol, nucleus and in the intermembrane space of the mitochondria [19]. It accounts for about 0.5–0.8% of the soluble protein in the human brain [20]. Mutations in this gene mainly display a dominant inheritance pattern, with complete or reduced penetrance. The collective evidence indicates that mutant SOD1 protein acts through the gain of a toxic property and not a loss of function [21,22,23]. To date, more than 180 *SOD1* mutations have been identified (ALS Online Genetic Database, ALSOD: http://alsod.iop.kcl.ac.uk/, accessed on 20 August 2021) [24]. The majority of them are missense mutations, with a small number of deletions and insertions resulting in truncated SOD1 polypeptides. Missense mutations have been documented all along the length of *SOD1* gene, with multiple amino acid substitutions involving a same codon. Overall, the clinical presentation of ALS patients carrying *SOD1* gene mutations does not differ significantly from sporadic ALS. Symptoms begin in the second half of the fifth decade (slightly earlier than SALS). The majority of *SOD1* cases present with spinal onset, and a significant percentage of them show pure LMN signs [25]. Most patients with *SOD1* mutations usually develop a rapidly progressive ALS, although some cases show a different phenotype. The disease phenotype may vary depending on the specific mutation and also among members of the same family. 

Since a toxic gain of function is considered the most likely pathogenetic mechanism triggered by *SOD1* mutations [18,19,20], a potential approach to therapeutic intervention consists in lowering the concentration of mutant SOD1 protein [26,27]. Gene therapy clinical trials are currently underway for ALS patients with *SOD1* mutations [28]. Tofersen, an antisense oligonucleotide (ASO) targeting *SOD1* messenger RNA transcripts (mRNAs), administered intrathecally, has been shown to be generally safe and tolerated [29], and able to decrease SOD1 concentrations in cerebrospinal fluid (CSF), reducing the rate of decline in *SOD1* patients, in particular at high dosage and in the fast-progressing group [30]. A phase-III study on safety and efficacy are currently ongoing, and ASO therapy seems to be a very promising approach to ALS caused by *SOD1* mutations.

We here report a novel missense mutation *p.V119M* (V118M according to the former nomenclature) in exon 4 of *SOD1* gene in two unrelated apparently sporadic ALS patient, both presenting with lower motor neuron phenotype but very different survival. We describe patients’ clinical features and evaluate the potential effect of this novel mutation on SOD1 protein structure and stability.

## 2. Materials and Methods

### 2.1. Patients’ Characterization 

ALS diagnosis was made accordingly to El Escorial Revisited criteria [31]. Family history was obtained through direct interviews. All patients gave their informed consent to participate in the study.

### 2.2. Molecular and Bioinformatics Analysis

Genomic DNA was extracted from peripheral blood using standard procedures. All the five exons and flanking regions of the *SOD1* gene were amplified by polymerase chain reaction (PCR) and analyzed by direct sequencing. Genetic analysis for the other major ALS-related genes (*C9orf72*, *TARDBP*, and *FUS*) was performed.

The effect of the newly detected SOD1 missense variant on protein structure or function was analyzed by using the following prediction programs: PROVEAN (Protein Variation Effect Analyzer) (http://provean.jcvi.org/index.php, accessed on 20 August 2021), PolyPhen (http://genetics.bwh.harvard.edu/pph2/, accessed on 20 August 2021) and Panther software (http://www.pantherdb.org, accessed on 20 August 2021).

Modelling of the *p.V119M* variant was performed using the Swiss model server (http://swissmodel.expasy.org/, accessed on 20 August 2021) [32] and the molecular visualization system Pymol (http://www.pymol.org/, accessed on 20 August 2021) from the crystal structure of the normal SOD1 protein (PDB2c9vA). Evaluation of model quality was performed by QMEAN global score.

## 3. Results

### 3.1. Case 1

The proband, a 78-year-old woman, at age 73 began to complain of distal and proximal progressive weakness of the left leg. Two years later the weakness spread to her right leg, leading the patient to be wheelchair confined. Neurological examination at diagnosis (age 78 years), 50 months from symptom onset, showed asymmetrical (more severe in the left side) weakness and wasting of the lower limbs, sporadic fasciculations of the thighs, with absent lower limbs tendon reflexes. Muscle strength and deep tendon reflexes in upper limbs were normal. Hoffmann and Babinski signs were absent. Sensory examination in all limbs, bulbar and respiratory functions were normal. The ALS Functional Rating Scale (ALSFRSr) score was 37. Extensive motor and sensory nerve conduction studies in all limbs revealed severe decrease of compound muscle action potential amplitudes in lower limbs as only abnormal finding. Electromyography (EMG) examination confirmed acute and chronic denervation signs in lumbar-sacral bilateral multiple myotomes and normal pattern in cervical, bulbar and thoracic muscular districts. Motor evoked potential (MEP) examination showed the sparing of central motor pathways. No autonomic or cognitive impairments were present. Brain and total spine magnetic resonance imaging (MRI) were negative for degenerative signs of pyramidal tracts or compressive abnormalities of lumbar-sacral nerve roots. Blood creatine kinase levels and CSF examination were normal. Diagnosis of pure lower motor neuron disease with flail leg phenotype was made. The DeltaFS at diagnosis scored 0.2/month showing a slow progression rate of the disease [33], confirmed by the subsequent clinical course. Indeed, the first respiratory dysfunction and bulbar symptoms occurred 20 and 27 months after diagnosis, respectively, whereas weakness in upper limbs appeared one year later. Non-invasive ventilation started at month 90 and death occurred 8 years and half after symptom onset, due to respiratory failure. Oral feeding and cognitive functions were spared throughout the course of the disease.

The patient, only child, had no family history of ALS, however their parents deceased at the age of 62 and 58 years and the possibility to develop the disease in an older age cannot be excluded. No anamnestic data are available about maternal and paternal grandparents. The patient has two sons, aged 53 and 43 years, and a daughter aged 51, who were clinically unaffected at the time of our examination. The daughter requested genetic test that did not reveal the presence of the c.355G>A variant in the *SOD1* gene.

### 3.2. Case 2

The proband, a 82-year-old woman, began to complain of progressive weakness of both lower limbs and reduction of hand motor dexterity 5 months earlier. Neurological examination showed symmetrical weakness and wasting of distal muscles of lower limbs with absent tendon reflexes, mild weakness of hand muscles with reduced tendon reflexes in upper limbs. Fasciculations were observed in muscles of thoracic and lumbo-sacral regions. Hoffmann and Babinski signs were absent. The use of two sticks was required for walking. Sensory examination in all limbs, bulbar and respiratory functions were normal. The ALSFRSr score was 39. Motor and sensory nerve conduction studies revealed reduction of compound muscle action potential amplitudes and mild slowing of motor conduction velocity in lower limbs. No alterations were detected in upper limbs. EMG examination showed acute and chronic severe denervation signs in lumbar-sacral and cervico-brachial bilateral multiple myotomes, and in thoracic paraspinal muscles, whereas muscles of cranial district had normal EMG pattern. MEP study showed absolute reduction amplitude of responses only in lower limbs with normal central conduction time in all districts. Autonomic and cognitive functions were spared. Brain and total spine MRI were normal. Blood creatine kinase levels were normal and CSF examination showed mild increase of albumine level. A diagnosis of pure lower motor neuron disease, with early involvement of axial muscles (paraspinal muscles), was made. The DeltaFS at diagnosis scored 1.8/month, suggesting fast progression rate of the disease [33]. Seven months later, the patient showed loss of ambulation and severe impairment of upper limbs with deterioration of daily living activities. Four months later she began to suffer from respiratory symptoms and non-invasive ventilation was started. Death occurred one year and half from disease onset, due to respiratory failure. Oral feeding and cognitive functions were spared throughout the course of the disease.

The patient, only child, had no family history of ALS. Her mother and father died in advanced age, of stroke and leukemia, respectively. No anamnestic data are available about maternal and paternal grandparents. The patient had a son, aged 51, and two daughters, aged 48 and 46 respectively, who were clinically unaffected during the illness of their mother. The son and the eldest daughter requested genetic test, which was negative in both for the c.355G>A variant in the *SOD1* gene. 

### 3.3. Molecular Analysis

**Figure 1:**
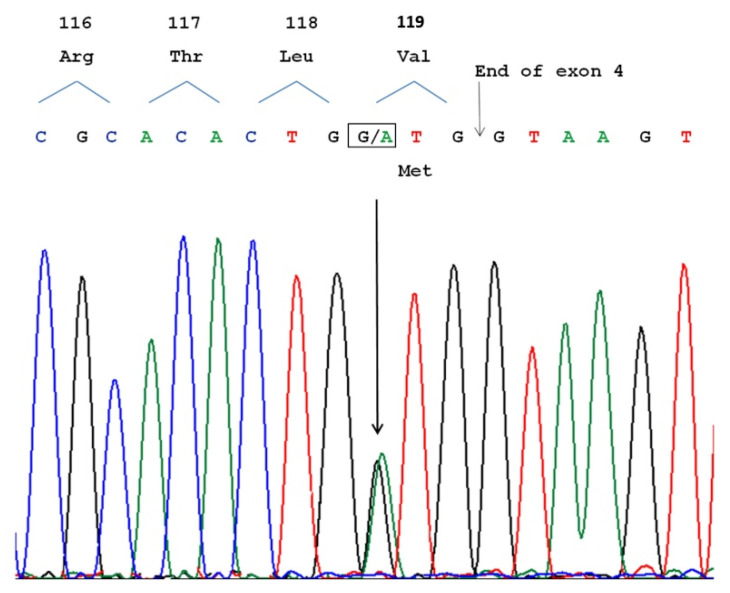
Automated sequence analysis of *SOD1* gene exon 4 showing the heterozygous mutation c.355G>A (arrow). The change GTG to ATG at codon 119, resulting in the substitution of methionine for valine in the SOD1 protein (p.V119M), is displayed in the box.

Molecular analysis showed a heterozygous variant c.355G>A in the *SOD1* gene (reference sequence NM_000454.4). The substitution of GTG to ATG in exon 4 determined a substitution of methionine for valine in SOD1 protein (p.V119M) (Figure 1). No alterations were detected in *C9orf72*, *TARDBP*, and *FUS* genes.

### 3.4. Bioinformatics Analysis

**Figure 2:**
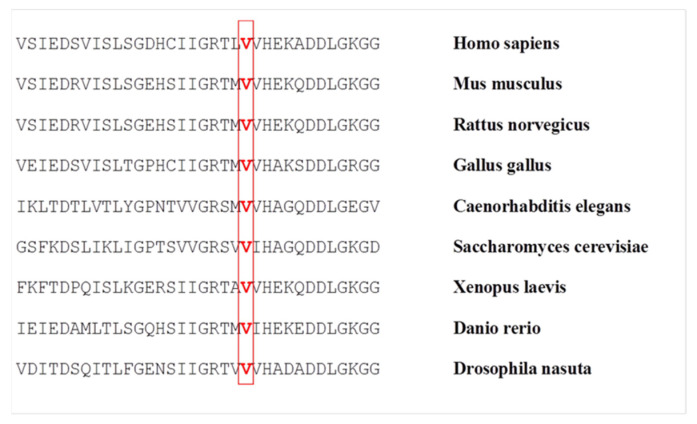
Protein alignment of the residues conserved across different species in SOD1 protein in the region around valine 119 (indicated in red).

Multi-species comparisons showed that the valine at codon 119 is a highly conserved residue among various species (Figure 2). A possible damaging effect of this amino acid substitution on protein structure/function was predicted by different *in silico* analyses: PROVEAN prediction software showed a score of −2.951 (cutoff threshold = −2.5) and classified the variants as “deleterious”; PolyPhen predicted the mutation as "probably damaging" with a score of 1.000; the pathogenicity predictor Panther revealed a SubSpec score of −5.199, which suggests a highly harmful mutation.

**Figure 3:**
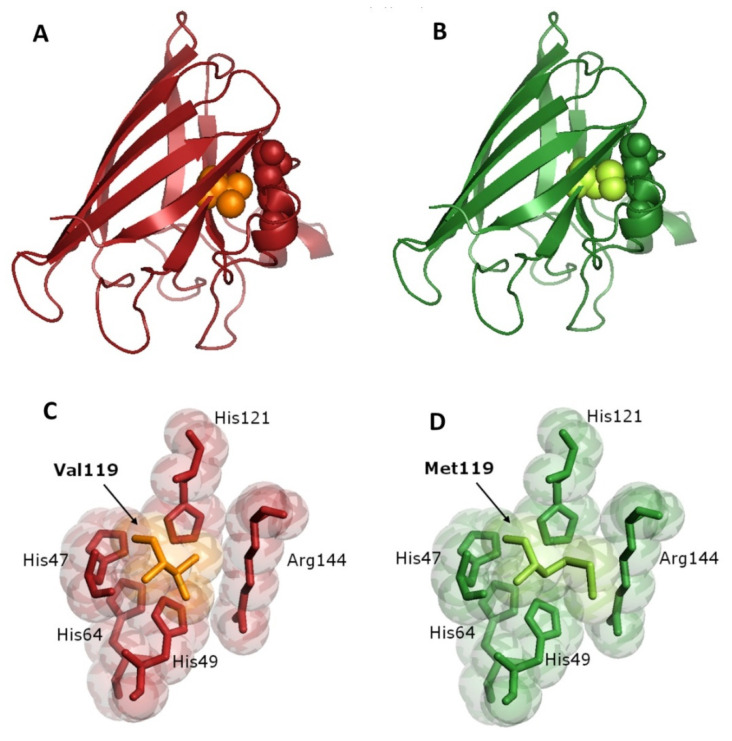
(**A**) Three-dimensional x-ray diffraction structure of wild type human SOD1 protein (PDB2c9vA); (**B**) Model of the SOD1 *p.V119M* mutation: spheres of the same color of the protein chain represent arginine 144, valine and methionine at position 119 are represented by orange/light green spheres, respectively; (**C**) Stereo image showing the amino acids constituting the copper binding site of wild type SOD1 protein (His47, His49, His64, His121 and Arg144) and Val119; (**D**) Stereo image showing the amino acids constituting the copper binding site of mutated *p.V119M* SOD1 protein (His47, His49, His64, His121 and Arg144) and the increased steric hindrance of Met119.

Modelization of the mutant SOD1 showed a destabilization of secondary structure in the amino acid sequence around the residue 119. The substitution of methionine for valine could alter the protein structure, in a region where it strictly interacts with the arginine at position 144 (Figure 3A,B) that, together to His47, His49, His64 and His121, is part of the copper-binding site in SOD1 protein. Thus, the *p.V119M* variant could modify the structure around the catalytic site (Figure 3C,D), with a consequent potential metal-binding alteration.

## 4. Discussion

Phenotypic heterogeneity for age at onset, disease duration, penetrance and clinical manifestations is not uncommon among patients with different *SOD1* mutations and also among members of the same families. Distinct clinical features can occasionally be correlated with specific *SOD1* mutations. For example, a uniform phenotype has been described for the p.G42S mutation [34], associated with an aggressive and rapidly progressive disease [18,35,36,37,38]. In this case, the clinical picture was characterized by spinal onset with early upper and lower motor neuron involvement, appearance of bulbar signs usually within one year from the onset, and death a few months later. Other mutations have been associated with significant LMN involvement without UMN signs. Among these, the phenotype of the p.A5V mutation is characterized by sudden symptom onset and relatively rapid disease progression, with a mean survival of usually 1 to 2 years. Muscle weakness can start in the limbs or in the bulbar muscles. LMN signs usually dominate the clinical presentation [35]. The p.A5T mutation, involving the same residue, is also associated with a similarly rapid disease course and LMN predominant phenotype [39]. In contrast, the p.G43C mutation has been related to a pure LMN clinical phenotype without bulbar involvement and more favourable prognosis with a median survival of 153 months [40].

In this study, we report a novel missense variant (*p.V119M*) in the *SOD1* gene in two unrelated Italian patients with apparently sporadic ALS. Clinical features of both patients were similar in regard to the site of onset (spinal in lower limb), the sparing of upper motor neuron, bulbar functions and cognitive status. However, they were very different regarding to progression rate of motor and respiratory impairments. 

The clinical features of Case 1, characterized by symptom restriction to the legs at 80 months from disease onset, and long survival, were consistent with the described course of the FL syndrome [8,10]. The FL variant is relatively rare, however it has been recently reported that *SOD1* mutations increase the frequency of this phenotype to a 3.5-fold [41].

Case 2, on the contrary, exhibited very fast progression rate and disease duration of 18 months. This disease progression may be similar to that reported for the p.A5V or p.A5T [26,30], however without involvement of bulbar muscles. This is not the typical phenotype of PMA, which usually shows a slower progression and a longer survival than classical ALS. However, when the paraspinal muscles are early involved, as showed in our patient, the disease is more aggressive and leads to a greater risk of respiratory care and therefore a worse prognosis [13]. Furthermore, as in typical ALS, also in PMA the diagnosis in older age (82 years in the current case) is usually associated with shorter survival [42].

Phenotypic variability among carriers of the same *SOD1* variant, also in the same family, is not new in ALS. However, considering the great difference in progression rate and survival between our patients, we cannot exclude the involvement of other unknown modulator genes or environmental factors, able to modify the disease course, especially in Case 2.

It was not possible to demonstrate the co-segregation of this novel mutation with the disease. However, some evidence supports this mutation as causative. The *p.V119M* variant is not present in the 1000 Genomes Project (www.internationalgenome.org, accessed on 20 August 2021), the largest public catalogue of human variation and genotype data. Different pathogenicity prediction software showed a highly probable damaging effect of this amino acid substitution on protein structure/function. Moreover, in the same codon a different missense mutation, resulting in the substitution of valine to leucine (p.V119L) has been previously identified in a Japanese sporadic ALS patient. In this case, the patient presented with weakness in her legs, early onset of bulbar symptoms, and rapidly progressive disease course [43,44]. It is not infrequent that different mutations involving the same amino acidic residue lead to very differing phenotypes. The p.C7S mutation, for example, results in a phenotype of variable progression and incomplete penetrance, whereas the p.C7G and p.C7F mutations are associated with a high penetrance and rapidly progressive disease course [45,46,47].

The Val119 amino acid residue is highly conserved in different species (Figure 2) and appears to be important for the proper structure and function of the SOD1 protein. In the quaternary structure of the enzyme, Val119 is located in the β-8 strand of the β-barrel. β-barrel mutations usually result in local perturbations able to alter the protein structure [48]. At the same time, the *p.V119M* mutation might act as a metal-binding mutation, that typically leads to altered SOD1-SOD1 interactions [49], since it interacts with the residues constituting the copper-binding site of SOD1. As shown in Figure 3, the increased steric hindrance of the methionine lateral chain modifies this part of the protein. This is particularly evident in the interaction with Arg144, an amino acid of critical importance for the catalytic activity [50], but also the stabilization of the protein structure. Thus, the presence of the *p.V119M* mutation might result in a failure of protein folding and a consequent genesis of off-pathway folding intracellular aggregates, toxic to motor neurons.

## 5. Conclusions

The identification of the novel *p.V119M* variant in the *SOD1* gene in two patients with pure lower motor neuron syndrome expands the spectrum of ALS phenotypes associated with *SOD1* mutations. Further studies on other ALS patients carrying the same mutation may confirm this genotype-phenotype correlation. A deeper knowledge of the different phenotypes associated with specific *SOD1* variants in ALS patients could be of great value on clarifying the nature of the disease and improving our understanding of pathogenic mechanisms. Last but not least, the identification of new pathogenic *SOD1* variants associated with ALS is nowadays of great relevance in the clinical management of patients with *SOD1* mutations, to give them opportunities for new therapeutic strategies based on antisense oligonucleotides targeting *SOD1* mRNAs. Furthermore, it may provide a potential target for more specific gene therapy and ultimately lead to personalized treatments. 

## Data Availability

The data presented in this study are available on request from the corresponding author. The data are not publicly available due to privacy restrictions.
